# LncRNA *LUCAT1* as a novel prognostic biomarker for patients with papillary thyroid cancer

**DOI:** 10.1038/s41598-019-50913-7

**Published:** 2019-10-07

**Authors:** B. Luzón-Toro, R. M. Fernández, J. M. Martos-Martínez, M. Rubio-Manzanares-Dorado, G. Antiñolo, S. Borrego

**Affiliations:** 1 Department of Maternofetal Medicine, Genetics and Reproduction, Institute of Biomedicine of Seville (IBIS), University Hospital Virgen del Rocío/CSIC/University of Seville, Seville, Spain; 2 Centre for Biomedical Network Research on Rare Diseases (CIBERER), Seville, Spain; 3 Endocrine Surgery Unit. General Surgery Department, University Hospital Virgen del Rocío, Seville, Spain

**Keywords:** Cancer epigenetics, Thyroid cancer

## Abstract

In recent years, long non-coding RNAs have emerged as a novel class of regulators of cancer biological processes. While they are dysregulated in many cancer types, little is known about their expression and functional profiles. This study has been focused on the determination of the role of a specific lncRNA in papillary thyroid cancer. Quantitative reverse transcription PCR was performed to detect the expression levels of 84 lncRNAs in 61 papillary thyroid carcinoma tissues and their adjacent non-tumor tissues. The highest fold-change was obtained for lung cancer associated transcript 1 *LUCAT1*, and thus, this study determines the expression and biological implication of lncRNA *LUCAT1* through different *in vitro* and *ex vivo* approaches in this tumor. *LUCAT1* was specifically located at the cell nucleus in tumoral regions of patient tissues. Furthermore, *LUCAT1* knockdown significantly reduced both cell proliferation and invasion *ex vivo* and induced cell-cycle arrest and apoptosis. These facts were corroborated by an enhanced expression of *P21*, *P57*, *P53* and *BAX*, and a reduced expression of *EZH2* and *HDAC1*. In addition, a significant decrease was observed on *DNMT1* and *NRF2* genes, helping to clarify the role of *LUCAT1* on PTC. Our study reveals the involvement of *LUCAT1* in PTC development, through acting in cell-cycle regulation, proliferation, epigenetic modifications through LUCAT1/ CDK1/ EZH2/ P57/ P21/ HDAC1/ DNMT1/ P53/ BAX axis and apoptosis, via extrinsic pathway activating caspases. These findings indicate that *LUCAT1* is maybe a potential therapeutic target and molecular biomarker for PTC.

## Introduction

Thyroid cancer is a rare tumor with a range of 2–20 cases/100,000 persons/year. It is the most common endocrine malignancy (90% of all endocrine neoplasms), and the leading cause of deaths of all endocrine tumors^1^. Papillary thyroid carcinoma (PTC) originates from the follicular cell epithelium of the thyroid. It presents as a solitary thyroid nodule, although in 35–45% of cases it can be associated with lymph node metastasis. Although much improvement on the treatment of PTC has been achieved, its prognosis remains very poor. Thus, the identification of the pathogenic mechanisms that trigger PTC is essential to improve the prognosis and therapies for this tumor.

The broad term long non-coding RNA (lncRNA) refers to a class of endogenous ncRNA transcript that is 200 nucleotides in length or larger and does not seem to contain a protein-coding sequence. Today it is known that some putative lncRNAs are translated into functional micropeptides, although they were misannotated as noncoding genes^2,3^. Even more, some lncRNAs are transcribed bidirectionally from promoters and enhancers^4^.

These molecules participate in transcriptional, epigenetic, or post-transcriptional regulation of gene expression, positively or negatively^5,6^. In human, there are from ~16,000 lncRNAs predicted by GENCODE^7^ to ~100,000 predicted by NONCODE^8^. These differences among both databases highlight either the huge number of lncRNA genes in the human genome and the growing need for steady lncRNA research.

An overwhelming amount of literature supporting the role of lncRNAs in cancer is emerging, where they can function as oncogenes or tumor suppressor genes. Two studies give further supporting evidence for the role of lncRNAs in cancer after the analysis of a huge group of clinical specimens^9,10^. Their outcomes reveal that, while in cancer the CpG island are hypermethylated, more than 1,000 lncRNAs are hypomethylated at their promoters. Consequently, lncRNAs are attractive and promising targets in the evaluation of cancer diagnosis, prognosis and distinct therapeutic strategies.

Since 2007 to date, many lncRNAs have been associated with PTC, although most of them have emerged in the last few years^11,12^. In this study, after a previous screening of several lncRNAs on PTC tissues, we focused on “lung cancer associated transcript 1” (*LUCAT1*). This lncRNA is located on chromosome 5 and comprises 4 exons and 3 introns but its function and molecular mechanism in PTC remains unclear. It was firstly described in the airway epithelium of cigarette smokers^13^ and to date has been associated with different tumors, which reinforces its importance as a prognostic value in cancer. In addition, it is considered as a potential prognostic biomarker and therapeutic target for each type of tumor with which it has been linked.

Two studies about ovarian cancer and overexpression of *LUCAT1* have been described and revealed that LUCAT1/miR-612/HOXA13 pathway modulates ovarian cancer progression^14,15^. It has been also detected in lung cancer, where *LUCAT1* epigenetically repress p21 and p57^16^, and decrease *DNMT1* and/or *DNMT3B* expression levels^17^. Some studies in colorectal cancer have obtained that *LUCAT1* induces cell cycle arrest and apoptosis by activating the ribosomal protein RPL40-MDM2-p53 pathway^18,19^.

Different studies have been performed on clear cell renal cell carcinoma, where *LUCAT1* was upregulated and it binds to polycomb PRC2 complex and suppress *P57* expression^20^. It is essential for proliferation and invasion and directly inhibit the expression of microRNA-495-3p^21^. *LUCAT1* also induced cell cycle G1 arrest in this tumor^22^. Regarding the role that plays *LUCAT1* in hepatocarcinoma, it directly sponges the onco-miR-181d-5p^23^. It promotes cell proliferation, migration and invasion through modulating miR-301b/STAT3 axis^24^ and it inhibits the phosphorylation of Annexin A2^25^. Finally, different associations of *LUCAT1* with other types of cancers have been also published, such as in glioma, where it regulates miR-375^26^ and in osteosarcoma, where it acts through miR-200c/ABCB1 pathway^27^. Furthermore, *LUCAT1* also acts on esophageal squamous cell carcinoma where it regulates the stability of DNMT1 leading to the formation and metastasis of this carcinoma^28^. *LUCAT1* and *CCNB1* had a positive relationship in regulating the progress of bladder cancer^29^. It was also found to present a differential expression on head and neck squamous cell carcinoma, together with other lncRNAs^30^ and it exerts an oncogenic function in cervical cancer by binding to miR-181a^31^.

All abovementioned studies clearly defined *LUCAT1* as an important biomarker in cancer prognosis. However, the molecular mechanism in thyroid cancer and particularly in PTC needs further investigation.

## Materials and Methods

### Patients and tissue samples

Sixty-one papillary thyroid tumor tissues and their corresponding adjacent non-tumor thyroid tissues were obtained from PTC patients undergoing surgical resection. All patients had total thyroidectomy. The samples were snap frozen in liquid nitrogen and stored at −80 °C. All the clinical data are compiled in Table 1. A written informed consent was obtained from all the participants for clinical and molecular genetic studies, after a full explanation of the purpose and nature of all procedures used. The study was approved by the Ethics Committee for clinical research in the University Hospital Virgen del Rocío (Seville, Spain) and complies with the tenets of the declaration of Helsinki.

**Table 1:** Clinicopathological features of the enrolled PTC patients. Correlation between LUCAT1 expression and clinicopathologic factors in PTC validated cohort. *P < 0.05 is considered statistically significant.

Parameter	Case	Total (n = 61)	*LUCAT1 e*xpression		*P (Fisher’s exact test)*
			Low	High	
*Age, years*					
	<45	35	1	34	<0.0001*
	≥45	26	15	11	
*Gender*					
	Male	20	8	12	0,1225
	Female	41	8	33	
*Primary tumor*					
	T1-T2	30	18	12	<0.0001*
	T3-T4	31	2	29	
*Lymph node metastasis*					
	N0	26	26	0	<0.0001*
	N1	35	6	29	
*Stage*					
	I,II	38	15	23	0,0167*
	III,IV	23	2	21	
*TNM*					
	M0	50	45	5	<0.0001*
	M1	11	1	10	

TNM: tumor-node-metastasis.

In addition, formalin-fixed paraffin-embedded (FFPE) tumor tissue samples from the same snap frozen samples, were used. All samples were from the University Hospital Virgen del Rocío-Institute of Biomedicine of Seville Biobank (Andalusian Public Health System Biobank and ISCIII-Red de Biobancos PT13/0010/0056).

### Screening by lncRNA PCR Array

Total RNA was obtained from tissues of our patients and commercial cell lines by using RNEasy Purification Kit (Qiagen), according to the manufacturer’s instructions. The RNA was quantified by Nanodrop (Invitrogen) and 1 μg of total RNA was reverse transcribed into cDNA using PrimeScript RT Reagent Kit (TaKaRa) to determine lncRNA expression levels, using *GAPDH* as reference gene. For lncRNA expression analysis, laboratory-verified SYBR^®^Green qPCR assays (RT^2^ lncRNA PCR Array, Qiagen) were used. Each plate contains 84 lncRNAs already associated with different cancer pathways (Supplementary Table 1). The quantitative real-time PCR (qRT-PCR) was performed at the 7900HT Fast Real-Time PCR System with the 384-Well Block Module (Applied Biosystems). All the reactions were carried out in triplicate. All the data were analysed by Applied Biosystems software and the relative expression levels of lncRNAs were determined by the equation 2^−ΔΔCt^. Two-tailed t-test was used to analyse differences between tumor tissues and their corresponding adjacent non-tumor thyroid tissues. A *P* < 0.05 was considered as a statistically significant difference.

To quantify the silencing level of *LUCAT1* in cell lines, the qRT-PCR was performed at 7500 Fast Real Time PCR System (Applied Biosystems) by using the primers provided by the manufacturer (Human LUCAT1, LPH16113A-200, Qiagen). All the reactions were carried out in triplicate.

### ViewRNA ISH Tissue 1-Plex Assay

For the *in-situ* detection of *LUCAT1*, the ViewRNA^TM^ ISH Tissue 1-Plex Assay (Affymetrix) on 5 μm FFPE tissue sections was used. The design of the probe of *LUCAT1* was based on the Refseq variant NR_103548 (http://www.ncbi.nlm.nih.gov/nuccore). The specificity of the signal was compared within each slide among the tumoral tissue and its normal paired tissue. The omission of the target probe set was used as a negative control. Images were captured by a TCS SP2 AOBS Spectral Confocal (Leica), using both 405 and 561 nm laser excitation lines, to compare normal *vs* paired tumoral tissue in each sample. 63xOil immersion objective was used. Images were obtained by photomultiplier, at 23 °C of temperature and processed by Leica Confocal Software and Multicolor Package.

### Cell lines and siRNA transfection

Human papillary thyroid cancer cell lines (BCPAP, 8505c) were obtained from Deutsche Sammlung von Mikroorganismen und Zellkulturen [DSMZ Cat# ACC-273 (established from a metastasizing papillary thyroid carcinoma) and ACC-219 (established from a papillary adenocarcinoma) respectively] and TPC1 cell line was a gift from Dr. Velázquez Henar (Autonomous University of Barcelona, Spain). All the cells were cultured in RPMI-1640 Medium (Invitrogen) supplemented with 10% fetal bovine serum at 37 °C in 5% CO_2_. The authentication method used by DMSZ is standardization of STR Profiling (ANSI eStandards Store, 2012).

For silencing assays, 250.000 cells were seeded in a 6-well plate and transfected when 70% confluent with siRNA1 at 20 μM. Initially, two different concentrations (5 or 20 μM) of two different siRNAs (siRNA1 and siRNA2; Silencer® Select siRNAs, ThermoFisher Scientific) were tested to decide the best one to use in all assays (siRNA1 at 20 μM). We also included a negative control siRNAs at the same concentrations and time points (si-NC; 24, 48 and 72 h), by using Viromer® BLUE Reagent (Viromer®) according to the manufacturer’s instructions. Then the cells were harvested for RNA extraction (Qiagen). Assays were performed three times.

### Cell proliferation

Cell Counting Kit-8 assay (CCK8) was used to quantify cell proliferation following manufacturer’s instructions. Briefly, 10.000 cells/96-well plates were silenced by siRNA1 (20 μm) during 24 and 48 h. Next day, each well was supplemented with the CCK-8 solution [2-(2-methoxy-4-nitrophenyl)-3-(4-nitrophenyl)-5-(2,4-disulfophenyl)-2H-tetrazolium, monosodium salt] which forms a formazan dye upon reduction in the presence of an electron mediator. After 4 h at 37 °C in 5% CO_2_ incubator, the absorbance (OD450 nm) was read in a microplate reader (BioRad). Data represent the mean ± SD from three independent experiments.

### Cell invasion assay

To determine cell invasive capacity after transfection of all cell lines with siRNA1 (20 μm) and negative control siRNA, transwell assay (ECMatrix Cell Invasion Assay, Millipore, 8.0-μm pores) was used, following the manufacturer instructions. Briefly, the kit utilizes ECMatrix™, a reconstituted basement membrane matrix of proteins derived from the Engelbreth Holm-Swarm mouse tumor. Cells (0.5 × 10^6^) in 500 μl medium with 10% FBS were placed into the insert. After 24 h incubation, non-invading cells as well as the ECMatrix gel were removed from the interior of the inserts and 500 μl of staining solution was added to the unoccupied wells of the plate. Images were acquired using a fluorescence microscope (BX61) with camera (DP72) (Olympus) and 10x objective. The number of migrated cells was quantified by dissolving stained cells in 10% acetic acid and the absorbance (OD560nm) was read in a microplate reader (BioRad). Data represent the mean ± SD from three independent experiments.

### Western blotting

For protein extracts, cells were seeded at a density of 250.000 cells/well and transfected with siRNA1 (20 μm) to be collected after 24 h using RIPA buffer (Sigma-Aldrich). The protein quality was quantified by Bradford method (ThermoFisher Scientific). Protein extracts were separated by 4–20% SDS-PAGE, equal amount of protein was transferred onto polyvinylidenefluoride (BioRad) membranes and incubated with the following primary antibodies: anti-P21 (Cell Signaling Technology, CST; Cat# 2947), anti-P57 (CST, Cat# 2557), anti-P53 (CST, Cat# 2524), anti-EZH2 (D2C9) (CST, Cat# 5246), anti-NRF2 (D1Z9C) XP (CST, Cat#12721), anti-HDAC1 (CST, Cat#34589), anti-CDK1 (abcam, [A17] ab18), anti-DNMT1 (abcam, [60B1220,1] ab13537), and anti-BAX (CST, Cat# 2772). Regarding the procaspase assay, the Procaspase Antibody Sampler Kit (CST, Cat#12742).

All primary antibodies were used at 1:1000 at 4 °C for 24 h, except for anti-Lamin (1:2000). After the incubation with secondary antibodies (anti-mouse IgG HRP Linked Antibody, Cell Signaling Technology, 1:2000; anti-rabbit IgG, HRP Linked whole Ab, Amersham, 1:20000), the bands were visualized by ECL (BioRad) and quantified on a ChemiDoc XRS + (BioRad) system by using ImageLab software. GAPDH served as internal control (anti-GAPDH, D16H11, CST, Cat# 5174). All assays were performed three times.

### Apoptosis and cell-cycle assays

All cell lines were transfected following our selected conditions (siRNA1, 20 μm, 24 h). Then, 1 × 10^6^ cells were collected and washed twice with ice-cold PBS. For apoptosis assay, cells were dual stained using FITC Annexin V Apoptosis Detection Kit I (BD Biosciences) according to the manufacturer’s protocol.

For cell-cycle analysis, cells were fixed with ethanol 70%, washed with PBS and then treated with RNAase A (100 ug/ul) and IP. Stained cells were immediately analysed using a flow cytometer (BD Biosciences). All assays were performed three times.

### Caspases assays

The detection of active caspase-3/7 and caspase-6, central effector caspases in apoptosis, in our three different silenced cells, was based on the bioluminescence chemistry commercially available as Caspase-Glo^®^ 3/7 reagent and Caspase-Glo^®^ 6 reagent (G8090 and G0970 respectively, Promega). Ninety-six well plates were seeded with 20,000 cells and incubated for 3 hours in free serum medium. After that, conditioned medium was substituted with the Caspase-Glo reagent following the manufacturer recommendations. Luminescence was measured with the Fluoroskan Ascent Microplate Reader (ThermoFisher Scientific) and results are reported as relative light units (RLU, background subtraction).

### Statistical analysis

The Student’s *t* test (two-tailed) was used to analyse all data. A *P* < 0.05 was considered statistically significant.

Levene’s test was used to assess the equality of variances for gender and age of patients in two groups (high and low *LUCAT1* expression), through t-test. A *P* < 0.05 was considered statistically significant.

## Results

### Screening phase of the study

The expression profiles of 84 lncRNAs, already associated with different cancer pathways, were determined in 61 tumoral and non-tumoral paired tissues by SYBR^®^Green qPCR assays. Thirty-five differentially expressed lncRNAs were detected in our samples (all adjusted *P* ≤ 0.05). From those altered lncRNAs, 30 were downregulated and 5 were upregulated (Table 2). It is important to highlight that 12 of these lncRNAs found (9 down and 3 upregulated) had been already described as associated with PTC, which reinforces the validity of our approach. Based on the greatest fold change (log2 = 6.45), the upregulated lncRNA *LUCAT1* was selected for further independent testing and validation.

### Specific *LUCAT1* location and expression at thyroid tissue in PTC patients

**Figure 1:**
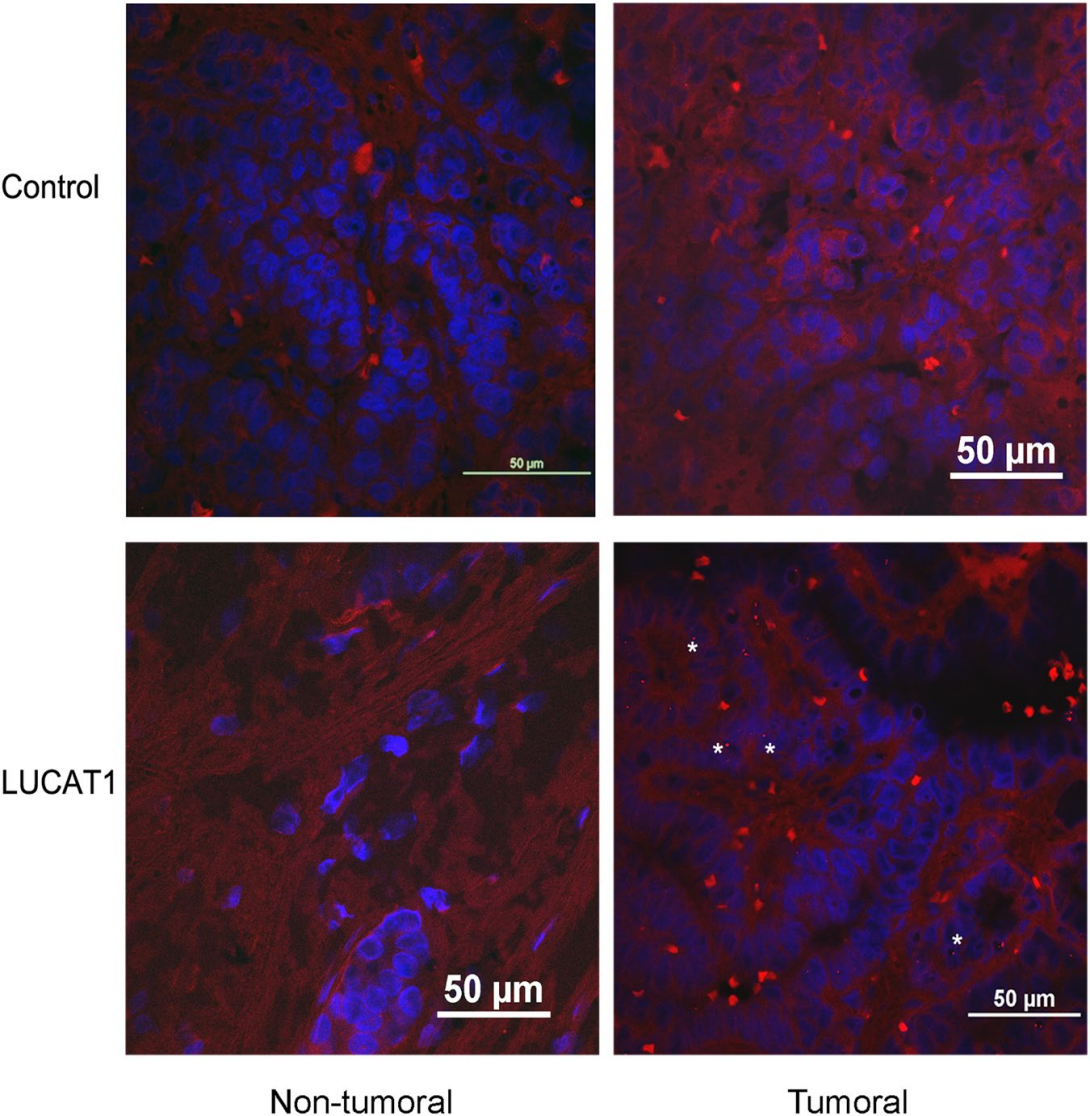
*LUCAT1* is specifically expressed at cell nucleus on tumoral regions from PTC tissues. The differences among the control samples hybridized without probe and samples incubated overnight with *LUCAT1* probe were analysed. Comparisons among tumoral and non tumoral regions from the same patient were realized. All samples (tumoral and non tumoral, with or without probe) proceed from the same the same PTC patient (serial FFPE slides, 5 μm). Different samples from PTC patients were analysed by double blind examination. The specific red punctuate was depicted (arrows). Magnifications: 63×. Scale bar: 50 μm.

The influence of *LUCAT1* on different crucial cell processes (proliferation, apoptosis, invasion and cell-cycle) led us to analyse its cell location. *In situ* hybridization analysis using a specific probe for *LUCAT1* was performed in FFPE samples from the same PTC patients where *LUCAT1* was found to be upregulated. Small red punctuates in tumoral regions of *LUCAT1* samples, but not in their normal regions, were detected. Furthermore, no specific red signal in any region of the control samples was found under confocal microscope after screening the whole sample. The red signal observed was weak, but specifically expressed in the cell nucleus (Fig. 1).

### *LUCAT1* expression and its association with clinical-pathologic features of patients

To further evaluate the prognostic value of *LUCAT1* in PTC, the association between clinicopathological features and *LUCAT1* overexpression, a univariate analysis by using the Fisher exact test was performed. Age, gender, primary tumor, lymph node metastases, stage and tumor-node-metastasis were analysed (Table 1). The 65% of patients analysed presented upregulation of this lncRNA, and from them, more than 50% showed a T3N1M1 stage, which suggests a positive correlation among overexpression of this lncRNA and an advanced stage of tumor in these patients.

**Table 2:** Aberrant lncRNAs in human PTC tissues: All significant lncRNAs obtained by qRT-PCR (7900HT Taqman system) through the RT^2^ lncRNA PCR Arrays in our cohort of tissues from PTC patients.

LncRNA	P-value	Ct (average)	Expression	Fold change
CAHM	0.0000166	28.45	downregulated	0.57
HAND2-AS1	0.0000023	31.93	downregulated	0.23
HEIH	0.0015059	26.76	downregulated	0.64
HULC	0.0205454	30.87	downregulated	0.7
KCNQ1OT1	0.0002485	27.31	downregulated	0.52
GAS5	0.0182714	26.10	upregulated	1.84
LUCAT1	0.0006995	28.75	upregulated	6.45
LINC00963	0.0064283	27.84	downregulated	0.75
LINC01233	0.0117869	31.51	downregulated	0.67
LINC00312	0.0032486	30.24	downregulated	0.54
MIR17HG	0.0033552	29.75	downregulated	0.7
NRON	0.0185571	30.52	downregulated	0,7
POU5F1P5	0.0016513	29.48	downregulated	0.65
PRNCR1	0.0002028	27.37	downregulated	0.73
RMRP	0.0247415	19.78	downregulated	0.77
SNHG16	0.0189111	26.48	downregulated	0.8
SPRY4-IT1	0.0019224	28.38	downregulated	0.7
SUMO1P3	0.0002431	28.30	downregulated	0.66
TUG1	0.0001961	25.35	downregulated	0.5
XIST	0.0004757	23.35	downregulated	0.63
ACTA2-AS1	0.0155455	32.35	downregulated	0.44
CCAT1	0.0050830	32.37	downregulated	0.36
TSIX	0.0054958	32.53	downregulated	0.62
BANCR(*)	0.0000345	32.30	downregulated	0.56
H19(*)	0.0266375	27.80	downregulated	0.53
MALAT1(*)	0.0017976	26.58	downregulated	0.28
MEG3(*)	0.0061960	30.59	downregulated	0.33
PTCSC1(*)	0.0000000	25.05	downregulated	0.46
PTCSC3(*)	0.0000000	25.74	downregulated	0.65
PVT1(*)	0.0000025	30.36	downregulated	0.5
PCAT1(*)	0.0019005	30.53	downregulated	0.65
TERC(*)	0.0000121	29.02	downregulated	0.5
LINC00152(*)	0.0003256	26.96	upregulated	2.69
MIR31HG(*)	0.0013127	29.47	upregulated	6.38
LINC00887(*)	0.0086052	31.48	upregulated	5.08

(*) Already described in association with PTC.

The t-test of Levene’s test revealed that the average age of onset of tumor was 17,35 years more for patients with low LUCAT1 expression *versus* those with LUCAT1 overexpression (IC95% 10,18; 24,52) in a significant manner (p < 0.001). These data suggest that *LUCAT1* might play as an unfavourable prognostic factor.

### Knockdown of *LUCAT1* impairs cell proliferation and leads to cell-cycle arrest

**Figure 2:**
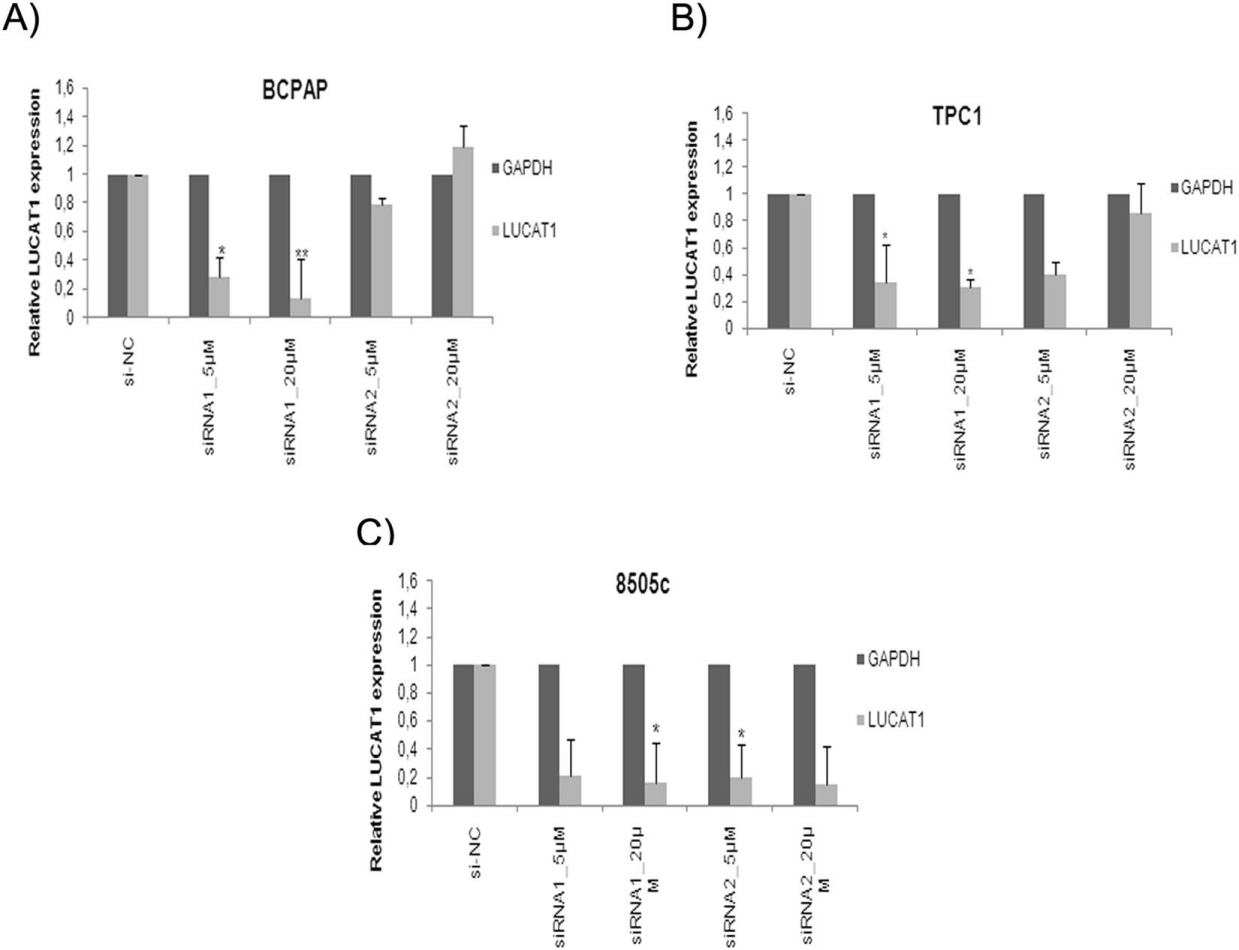
Silencing of *LUCAT1* by siRNAs. The expression levels of *LUCAT1* were detected by qRT-PCR in (**A**) B-CPAP, (**B**) TPC1 and (**C**) 8050c silenced cells with control siRNA (siNC) and *LUCAT1* siRNAs (1 and 2) at 24 h and two concentrations (5 and 20 μm). Data represent the mean ± SD from three independent experiments. *P < 0.05; **P < 0.01; ***P < 0.001.

The qRT-PCR results revealed that *LUCAT1* expression levels were significantly reduced (86.24% on BCPAP; 68,5% on TPC1 and 88.5% on 8505c) after 24 h transfection with siRNA1 at 20 µM, in comparison with si-NC (Fig. 2). No significant differences were found at 48 h and 72 h (Supplementary Table 2).

**Figure 3:**
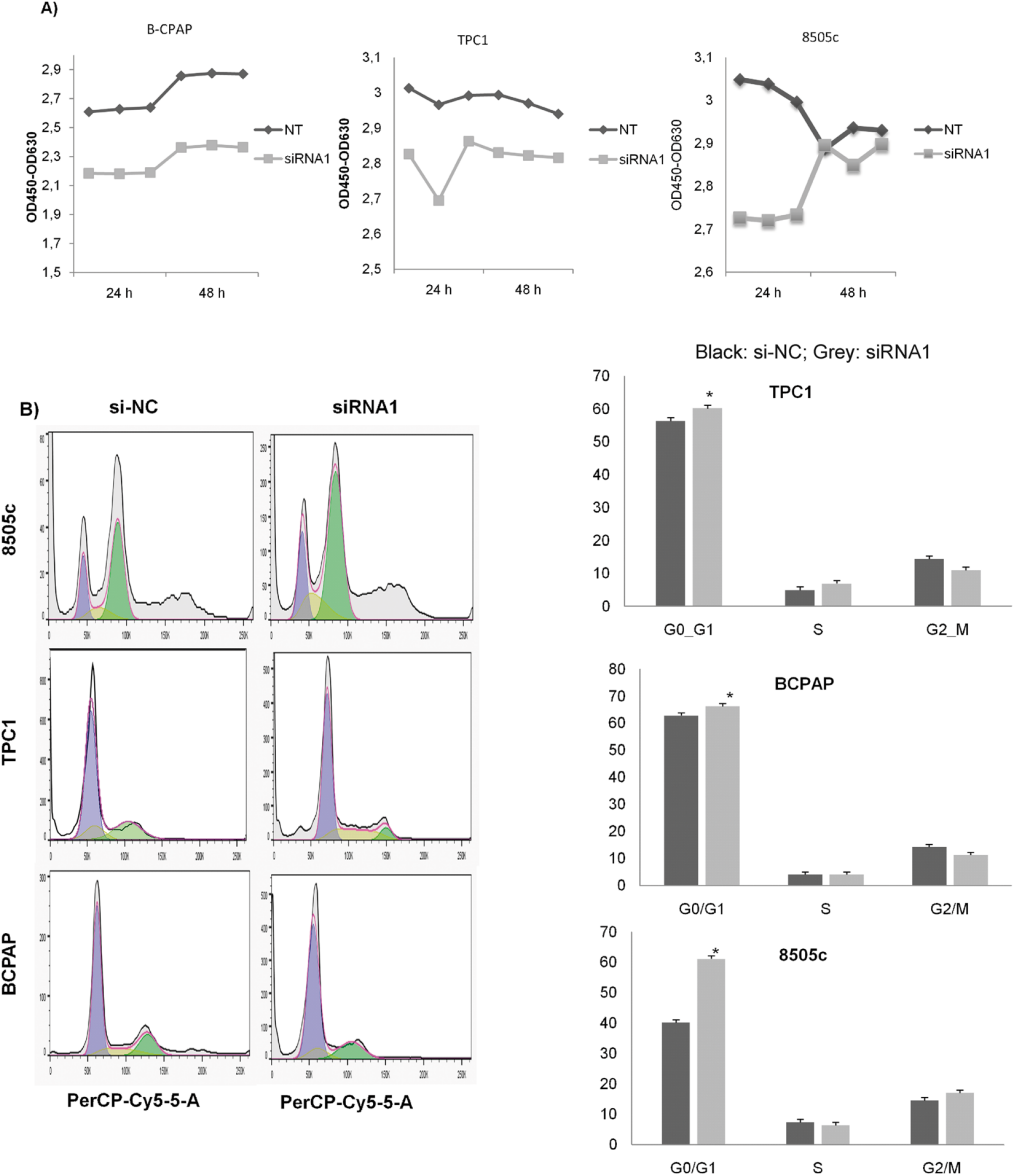
Knockdown of *LUCAT1* inhibited cell proliferation and induced a cell cycle arrest. (**A**) Representation of the effects on the proliferation (OD450nm) after transfection with *LUCAT1* with siRNA1 (20 μm) at 24 h and negative control (siNC). (**B**) Flow cytometry analysis of cell cycle 24 h after transfection with siRNA1 (20 μm) in all three cell lines. Data represent the mean ± SD from three independent experiments. *P < 0.05; **P < 0.01; ***P < 0.001.

After the knockdown of *LUCAT*, a significant reduction in cell proliferation (Fig. 3A) and cell G1 phase arrest (Fig. 3B) was detected in all cell lines analysed, after performing CCK8 assays and flow cytometry respectively. These findings indicate that *LUCAT1* may play an important role in PTC development.

### Knockdown of *LUCAT1* induces cell apoptosis and impairs cell invasion

**Figure 4:**
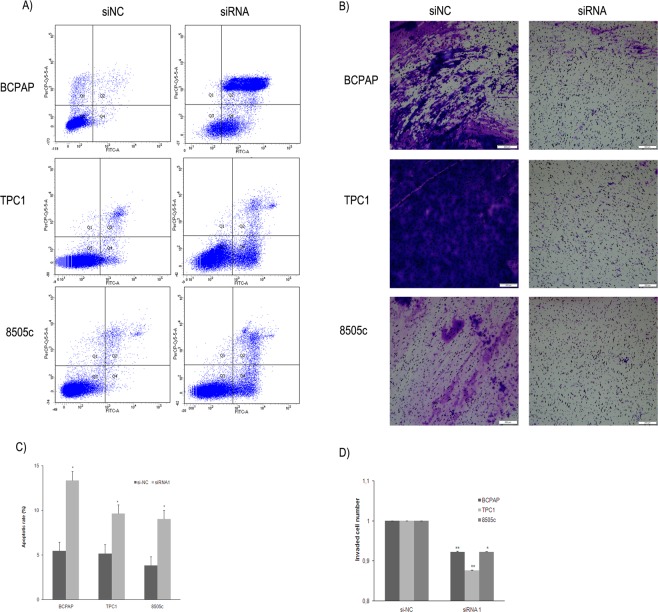
Inhibition of *LUCAT1* expression induced apoptosis and inhibited cell invasion. (**A**) Apoptosis was measured, by flow cytometry using PI/Annexin V staining, on silenced cells (siRNA1, 20 μm, 24 h). (**B**) Quantification of three independent replicates assays of apoptosis. (**C**) Representative images (4x magnification, 500 μm) of the polycarbonate membranes showing a significant reduction of invasion ability after transfection to silenced *LUCAT1*, as well as with negative control (siNC). Cells that migrated through the membrane were quantified by dissolving stained cells in 10% acetic acid for colorimetric reading of OD560nm. (**D**) Quantification of three independent replicates assays of cell invasion. Data represent the mean ± SD from three independent experiments. *P < 0.05; **P < 0.01; ***P < 0.001.

To further determine the role of *LUCAT1* in apoptosis, flow cytometry analyses were performed on our three PTC cell lines, and the outcomes showed that silenced *LUCAT1* would induce cell apoptosis in all cell lines studied (Fig. 4A). In addition, transwell assays unveiled a significant impairment of the invasive ability of these transfected cells (Fig. 4B). These data suggest that *LUCAT1* might be playing a relevant role in PTC progression through regulating cell apoptosis and invasion.

### Knockdown of *LUCAT1* induces caspase-8 activation and cleavage of nuclear downstream targets

**Figure 5:**
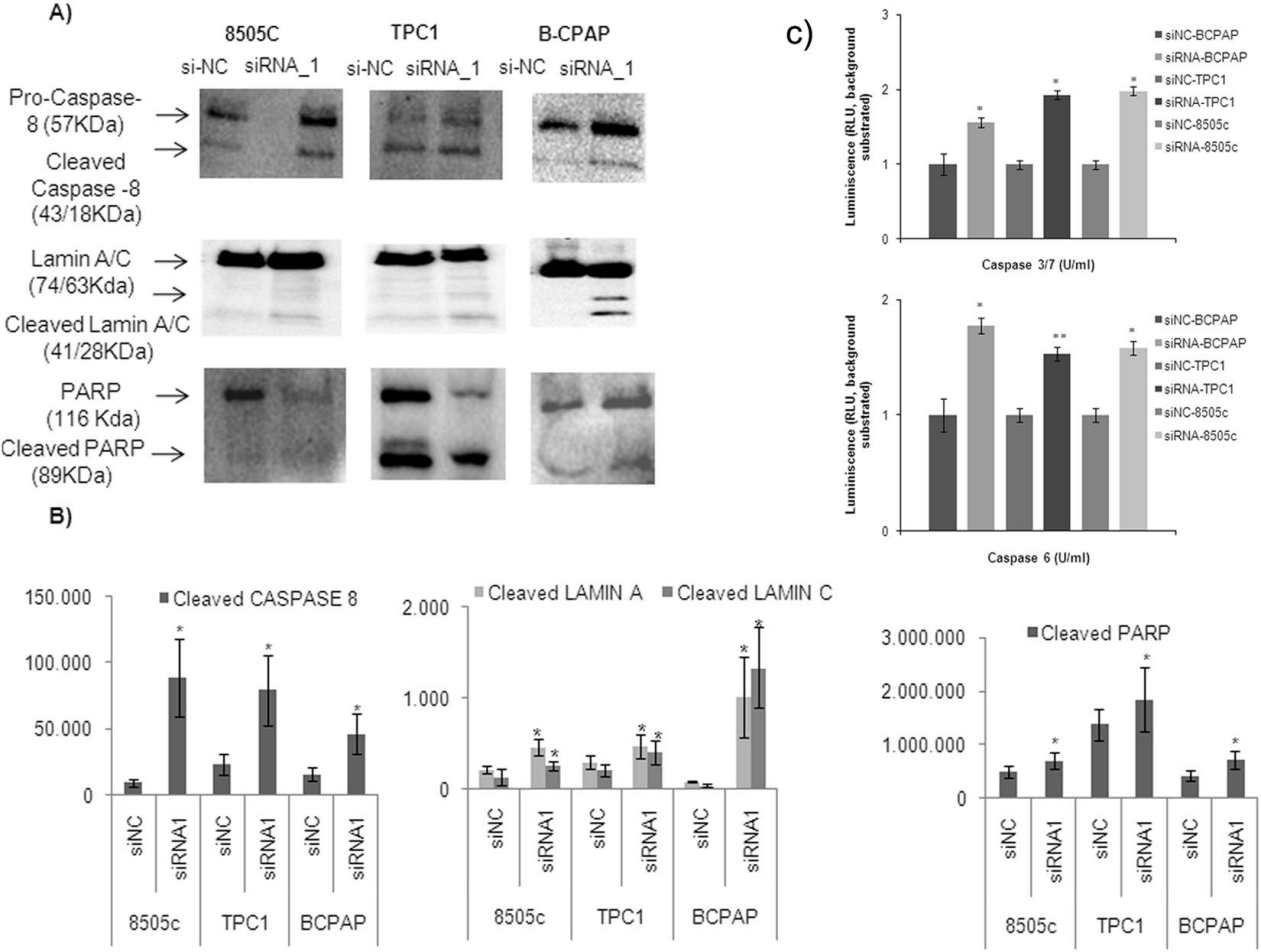
Regulation of apoptosis via extrinsic pathway activating caspases by *LUCAT1*. (**A**) Western Blotting analysis of cleaved (activated) caspase 8 and dowstream targets (Lamin and PARP) in BCPAP, TPC1 and 8505c cell lines and (**B**) Quantification by quimioluminiscence of three independent experiments was performed by ImageLab software. (**C**) Luminiscent assay to measure the activation of caspases –3, 6 and −7 in the three cell lines studied. *P < 0.05; **P < 0.01; ***P < 0.001.

To further explore the implication of *LUCAT1* in apoptosis, the initiator caspase-8 and 9 were analysed by Western Blot. A significant cleavage was detected for caspase-8, suggesting that the extrinsic pathway was followed. Following caspase-8 activation, downstream activation of effector caspases (3, 6 and 7) was analysed by luminescence. All of them were active and able to cleavage downstream nuclear targets as Lamin A/C and PARP (Fig. 5).

### Potential role of *LUCAT1* as a tumor activator

**Figure 6:**
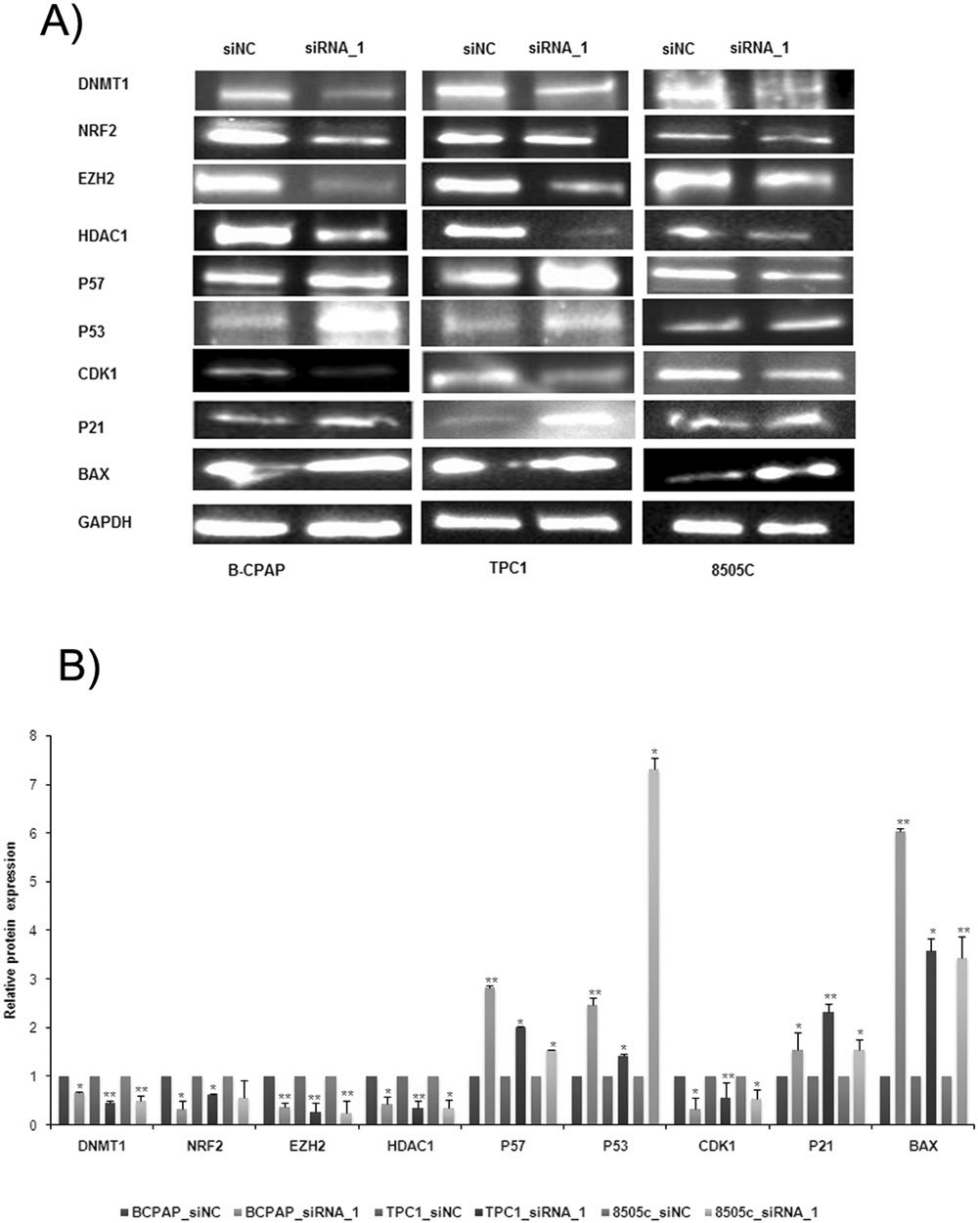
Different targets regulated by *LUCAT1*. (**A**) Western Blotting analysis of the expression of different proteins related with proliferation, apoptosis, cell cycle and epigenetic mechanisms. Blots were cropped either from the different parts of the same gel or from different gels. (**B**) Quantification by quimioluminiscence of three independent experiments was performed by ImageLab software. *P < 0.05; **P < 0.01; ***P < 0.001.

**Figure 7:**
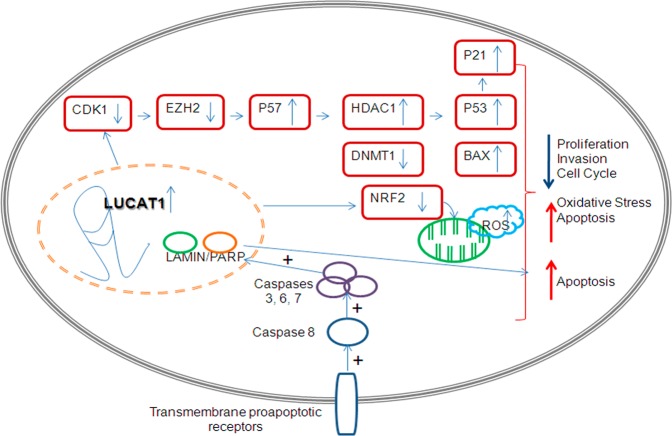
A schematic diagram depicting the possible model of *LUCAT1* action on PTC. A hypothetical model of action is proposed for *LUCAT1*.

Previous targets described for *LUCAT1*, together with other ones related with cell proliferation, differentiation and epigenetic regulation (HDAC1) and cell-cycle and apoptosis (P53, BAX and CDK1) were checked in our model through Western Blot. *P21*, *P57*, *P53* and *BAX* were significantly overexpressed while *EZH2*, *DNMT1*, *CDK1*, *HDAC1* and *NRF2* (this gene was not significantly downregulated in 8505c cell line), were significantly downregulated (Fig. 6A). After quantification of three independent experiments, the significant alterations on the protein expression levels were confirmed (Fig. 6B). A hypothetical model of action of *LUCAT1* is proposed (Fig. 7).

These results reinforce the potential role of *LUCAT1* as a transcriptional regulator by impairing cell proliferation and invasion, inducing apoptosis though extrinsic pathway activating caspases and leading to cell-cycle arrest.

## Discussion

*LUCAT1* has been found to be implicated in different biological processes in a broad variety of cancers, which reinforces its potential oncogenic role, but, to our knowledge, this is the first study analysing *LUCAT1* and its role in PTC development.

It is important to discern the potential association between a given lncRNA and clinical features of the patients evaluated, because it can be highlighted as an interesting molecular biomarker for a particular cancer type^32,33^. In our case, around 50% of patients presented upregulated *LUCAT1* and an advanced tumor stage, suggesting that it could be implicated in some mechanisms leading to the progression of PTC.

*LUCAT1* is specifically located in the cell nucleus of the tumoral regions, as it has been confirmed in renal carcinoma^22^. This fact suggests that it could regulate gene transcription^28,34^.

Furthermore, our outcomes indicate that *LUCAT1* might induce cell proliferation by regulating the cell-cycle, alike to other potential oncogenic lncRNAs^35^.

Unrestricted cell proliferation is a hallmark of cancers and is usually evoked by alterations in cyclin-dependent kinases (CDKs) activity^36^. The CDK1 protein is essential to entry into S-phase and mitosis^37^. Thus, *CDK1* knockdown hindered G1/S progression. A significant reduction of CDK1 was detected in all the cell lines tested, which supports the implication of *LUCAT1* in cell-cycle, supporting previous evidences on different carcinomas^16,22^.

There is a signalling link between CDK1 and EZH2, with a relevant role in cancer cell invasion^38^.

The downregulation of *EZH2* observed in our cell lines, could be produced by the reduced expression of CDK1 after *LUCAT1* knockdown that thus promotes the aberrant cell transformation to cancer cells. P57 is a direct target of EZH2 and, in fact, previous evidences revealed that *LUCAT1* epigenetically impaired the *P21* and *P57* expression^16^. Interestingly, *P21*, when localized specifically to the nucleus regulates cell proliferation and differentiation^39^.

In the current study, an increased expression of both *P21* and *P57* and a downregulation of *EZH2* would reinforce the role of *LUCAT1* on proliferation and cell-cycle regulation.

Recent evidences in PTC have confirmed that recruitment of polycomb repressor complexes PRC2 (with *EZH2*) and histone deacetylase *HDAC1/2* is needed for transcriptional repression and pro-proliferation through *P57*^40^. Both histone deacetylation (supported by HDACs)^41^ and DNA methylation (by DNA methyl transferases, DNMTs)^42^ are two relevant gene-silencing mechanisms which accelerate the progression of many cancers. *DNMT1* and *HDAC1* are known to be upregulated in PTC^43,44^.

Distant metastases are one of the main factors for overall survival in PTC^45^. Based on this concept, the invasion capacity and apoptosis of cells were tested. It is worthy to mention that *P53* together with *P21* produces a cell cycle G1 phase arrest^46^. In our cell model, a significant P53 and BAX proteins levels increase was detected, suggesting the implication of *LUCAT1* in this mechanism.

In this manner, *LUCAT1* is proposed to be related with PTC progression through LUCAT1/CDK1/EZH2/P57/P21/HDAC1/DNMT1/P53/BAX axis, suggesting its role as tumor activator in PTC, as it has been reported in other type of cancers^16,28^.

Finally, searching for a pathway involved on the development of PTC, and based on previous studies^13,28^, the expression level of *NRF2* was analysed. It has been described that the NRF2 pathway is commonly activated in PTC and its knockdown significantly decreased viability of PTC cell lines^47^. *NRF2* is implicated in cell survival through the regulation of the reactive oxygen species level^48^. Then, knockdown of *LUCAT1* reveals reduced *NRF2* levels, suggesting that NRF2 could be regulated by *LUCAT1* and both act on PTC progression.

Collectively, our study highlights *LUCAT1* as a new prognosis-related biomarker associated with PTC, through the LUCAT1/CDK1/EZH2/P57/P21/HDAC1/DNMT1/P53/BAX axis and with the influence of other mechanisms, such as increase of oxidative stress through the downregulation of the antioxidant gene *NRF2*.

## Supplementary information

Supplementary Information

Supplementary Table 1

Supplementary Table 2

## Data Availability

All data generated and/or analysed during this study are available from the corresponding author on reasonable request.
