# Supplementary material for: LncRNA LUCAT1 as a novel prognostic biomarker for patients with papillary thyroid cancer
Source: Sci Rep. 2019 Oct 7;9:14374. doi: 10.1038/s41598-019-50913-7 (PMC6779763; doi:10.1038/s41598-019-50913-7)
Supplement: Supplementary file 1 — Supplementary Information [file 41598_2019_50913_MOESM1_ESM.pdf]

Supplementary Information

LncRNA LUCAT1 as a novel prognostic biomarker for patients with papillary thyroid cancer.

Luzón Toro B, Fernández RM, Martos-Martínez JM, Rubio-Manzanares-Dorado M, Antiñolo G, Borrego S.

Supplementary Figure 1: All unprocessed original scans for all of the blots presented in figure 5.

Pro-Caspase 8 (18/43/7Kda)

8505C: siNC, siRNA-1 | TPC1: siNC, siRNA-1

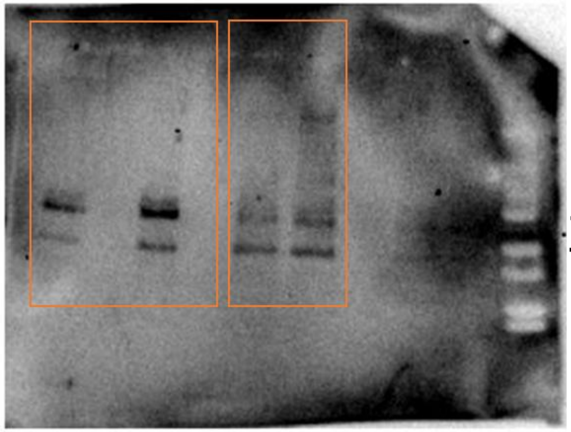

Caspase 8 (57 KDa)

Cleaved Caspase 8 (43/18KDa)

— 75 KDa
— 50 KDa

BCPAP: siRNA-1, siNC

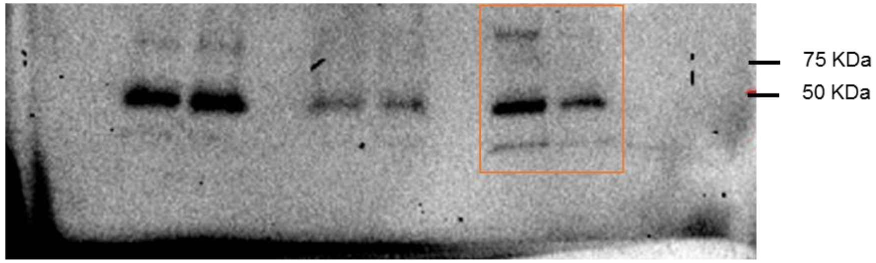

— 75 KDa
— 50 KDa

Lamin A/C (A:74 Kda; C:63 Kda)

8505C: siNC, siRNA-1 | TPC1: siNC, siRNA-1

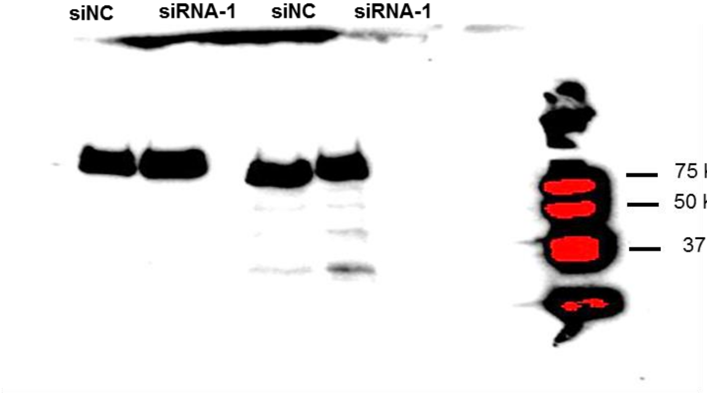

Lamin A/C (74/63 Kda)

Cleaved Lamin A/C (41/28 Kda)

— 75 KDa
— 50 KDa
— 37 KDa

BCPAP: siRNA-1, siNC

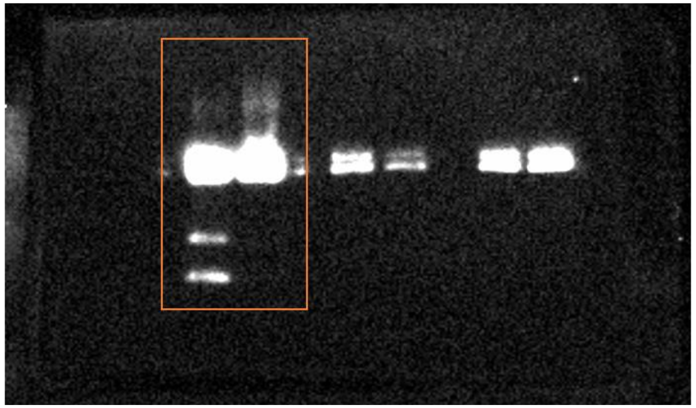

PARP (116/89 Kda)

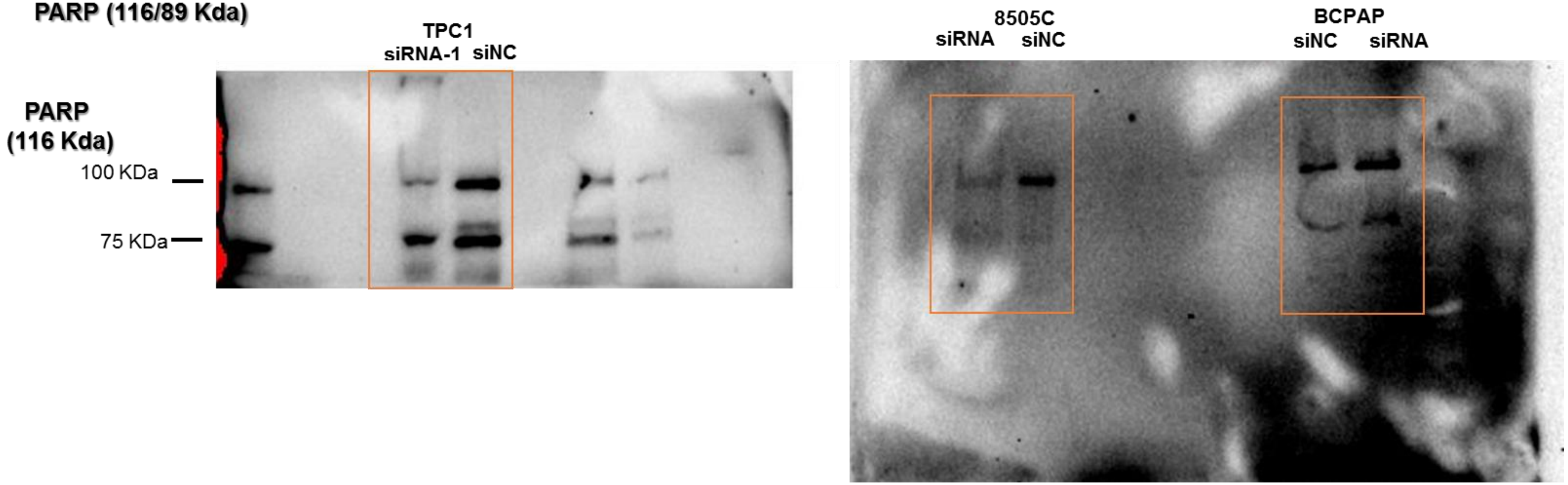

Supplementary Figure 2: All unprocessed original scans for all of the blots presented in figure 6.

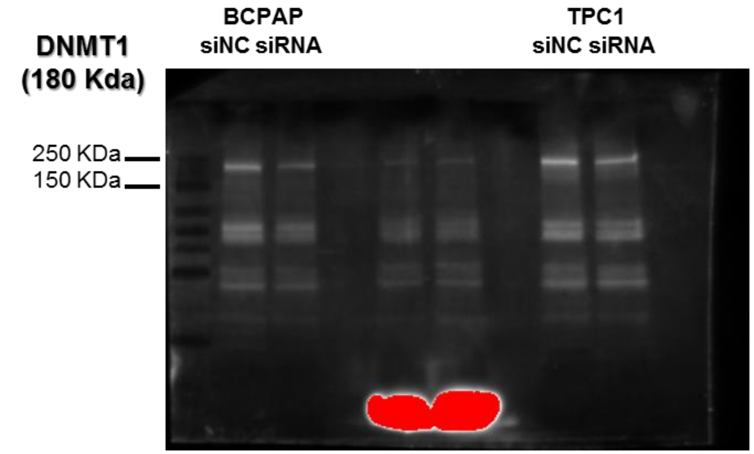

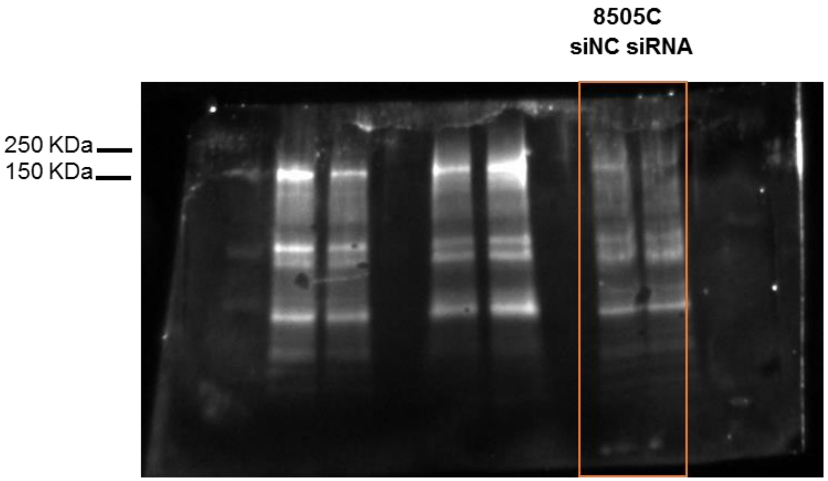

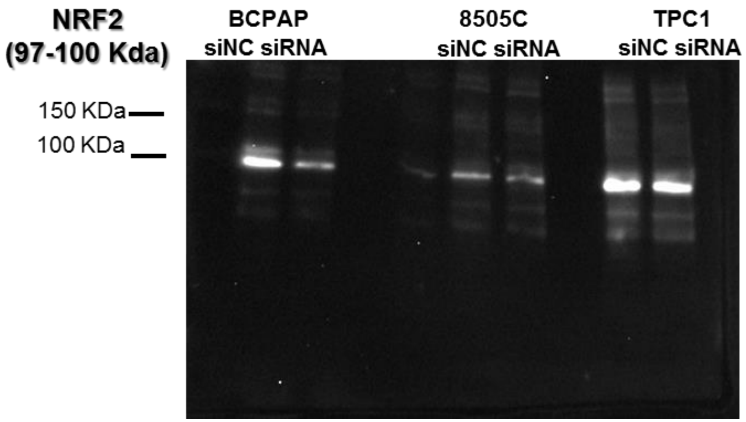

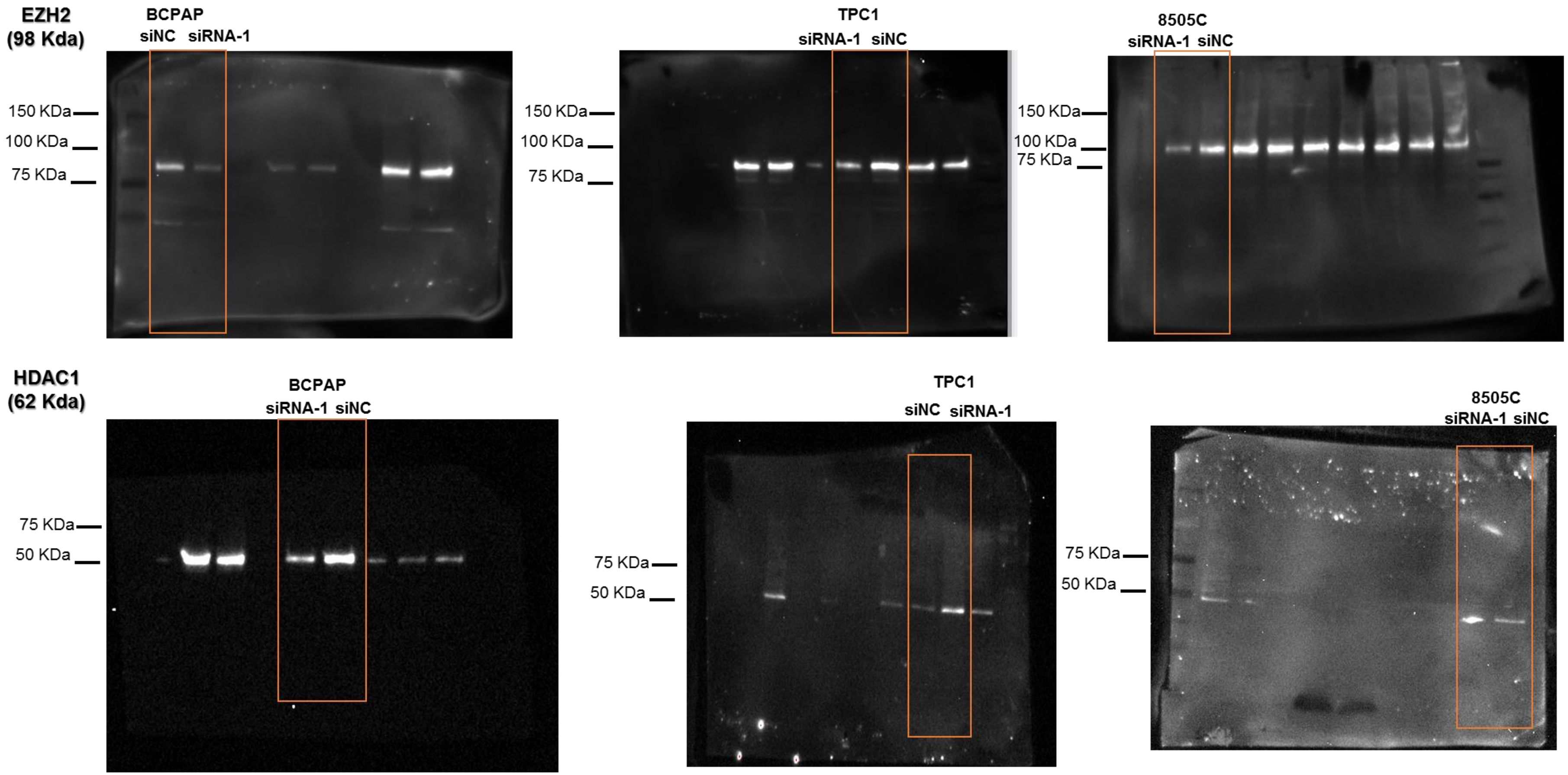
EZH2
(98 Kda)
BCPAP
siNC siRNA-1
150 KDa
100 KDa
75 KDa
TPC1
siRNA-1 siNC
150 KDa
100 KDa
75 KDa
8505C
siRNA-1 siNC
150 KDa
100 KDa
75 KDa
HDAC1
(62 Kda)
BCPAP
siRNA-1 siNC
75 KDa
50 KDa
TPC1
siNC siRNA-1
75 KDa
50 KDa
8505C
siRNA-1 siNC
75 KDa
50 KDa

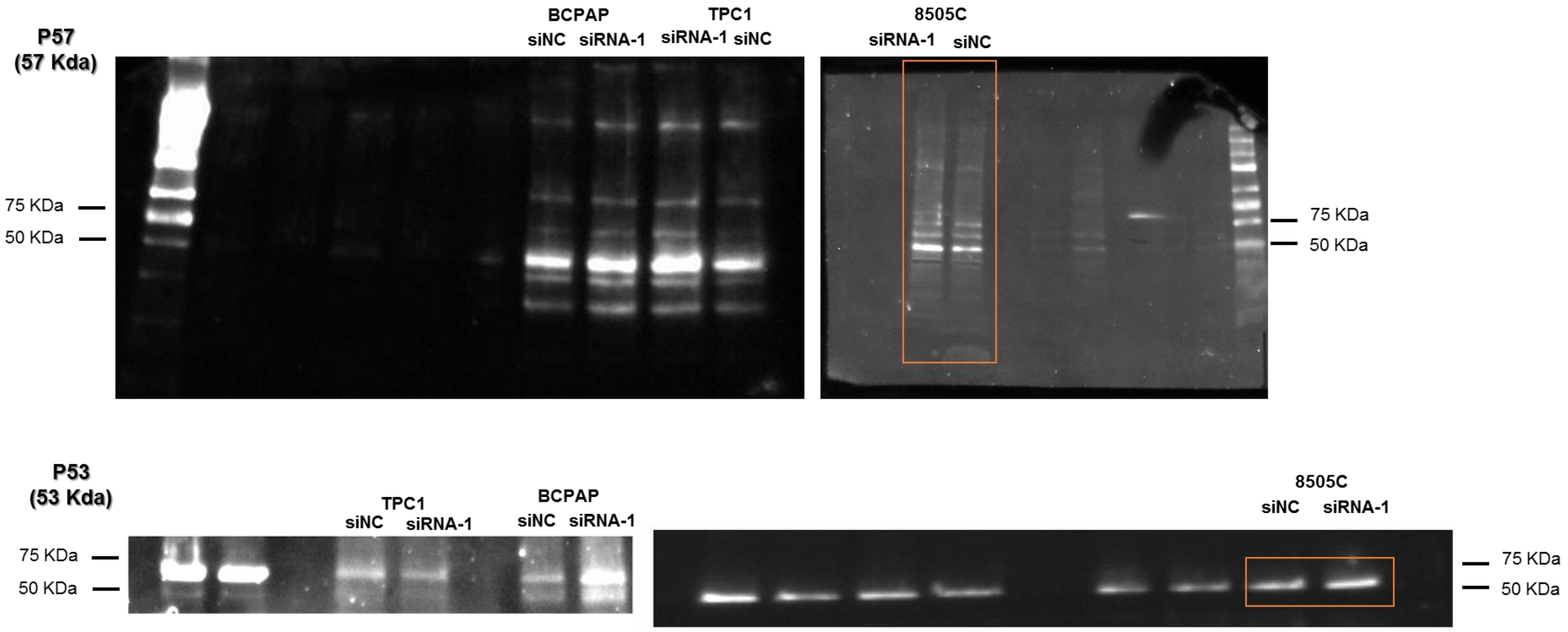
P57
(57 Kda)
BCPAP
siNC siRNA-1
TPC1
siRNA-1 siNC
8505C
siRNA-1 siNC
75 KDa
50 KDa
75 KDa
50 KDa
P53
(53 Kda)
TPC1
siNC siRNA-1
BCPAP
siNC siRNA-1
8505C
siNC siRNA-1
75 KDa
50 KDa
75 KDa
50 KDa

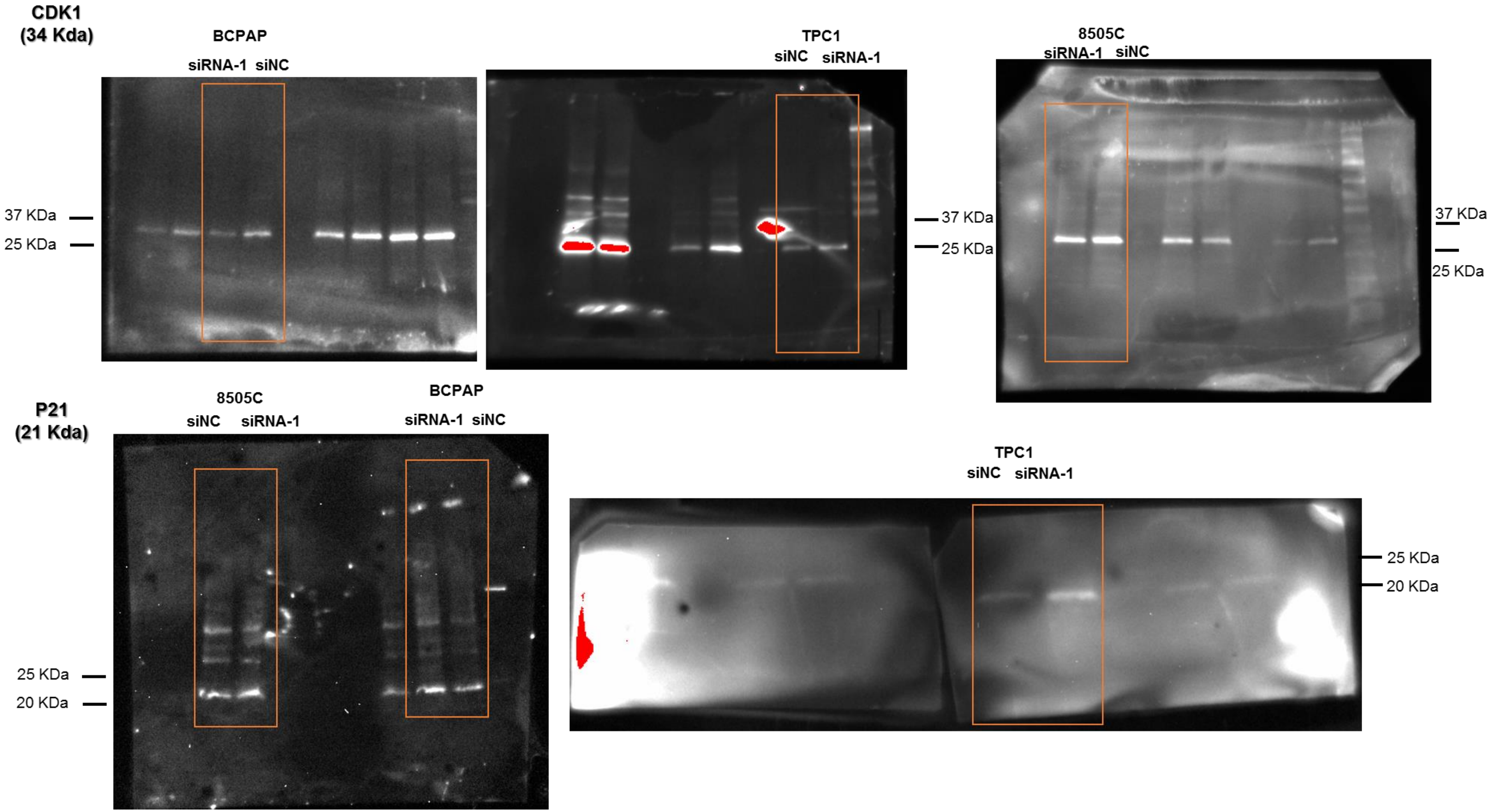

CDK1
(34 Kda)
BCPAP
siRNA-1 siNC
37 KDa
25 KDa
TPC1
siNC siRNA-1
37 KDa
25 KDa
8505C
siRNA-1 siNC
37 KDa
25 KDa
P21
(21 Kda)
8505C
siNC siRNA-1
BCPAP
siRNA-1 siNC
25 KDa
20 KDa
TPC1
siNC siRNA-1
25 KDa
20 KDa

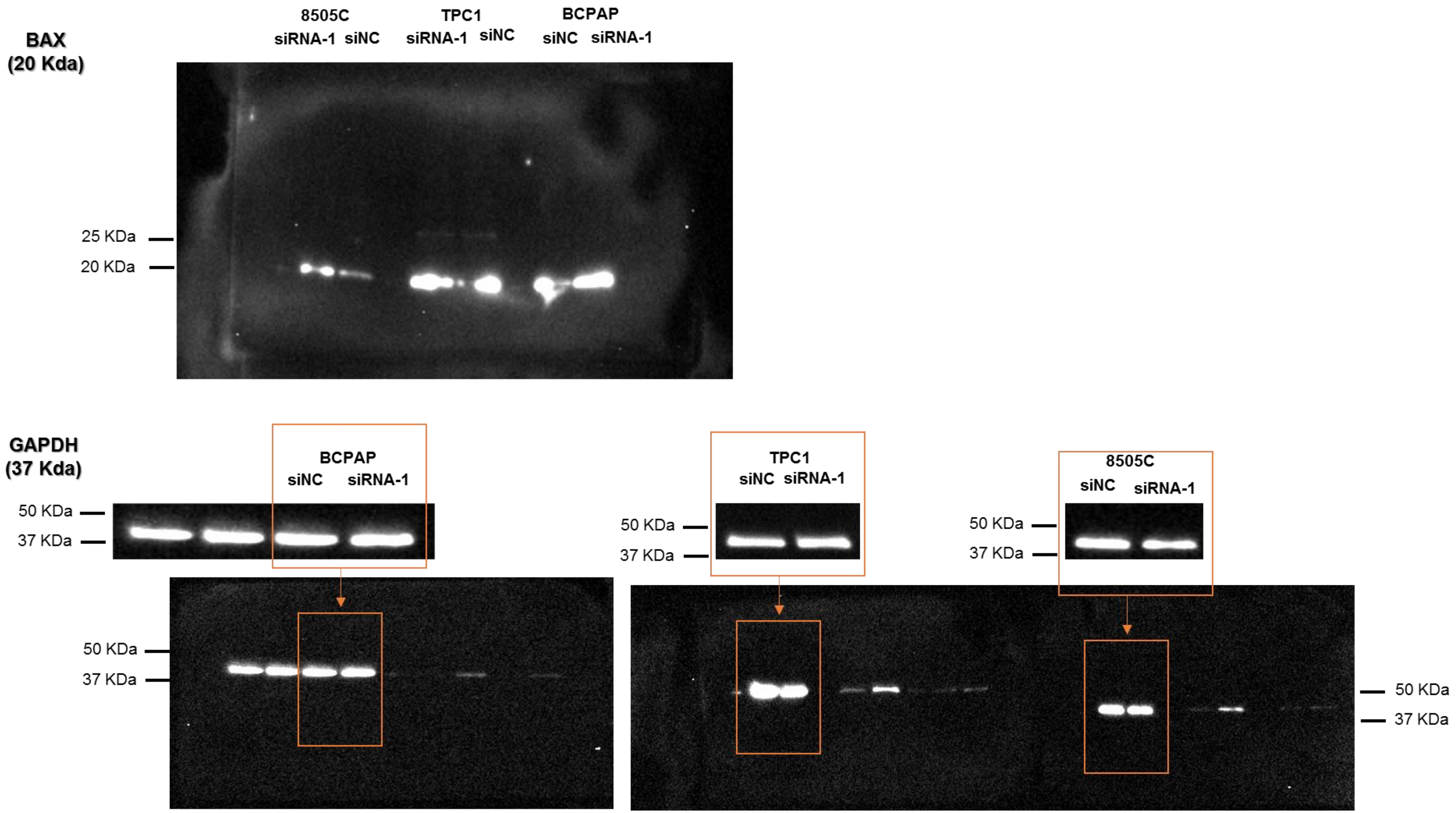
BAX
(20 Kda)
8505C
siRNA-1 siNC
TPC1
siRNA-1 siNC
BCPAP
siNC siRNA-1
25 KDa
20 KDa
GAPDH
(37 Kda)
BCPAP
siNC siRNA-1
50 KDa
37 KDa
TPC1
siNC siRNA-1
50 KDa
37 KDa
8505C
siNC siRNA-1
50 KDa
37 KDa
50 KDa
37 KDa
50 KDa
37 KDa
