# Supplementary material for: LncRNA LUCAT1 as a novel prognostic biomarker for patients with papillary thyroid cancer
Source: Sci Rep. 2019 Oct 7;9:14374. doi: 10.1038/s41598-019-50913-7 (PMC6779763; doi:10.1038/s41598-019-50913-7)
Supplement: Supplementary file 2 — Supplementary Table 1 [file 41598_2019_50913_MOESM2_ESM.pdf]

LncRNA *LUCAT1* as a novel prognostic biomarker for patients with papillary thyroid cancer.

LncRNA LUCAT1 as a novel prognostic biomarker for patients with papillary thyroid cancer.

Luzón Toro B[1,2], Fernández RM[1,2], Martos-Martínez JM[3], Rubio-Manzanares-Dorado M[3], Antiñolo G[1,2], Borrego S[1,2].

[1]Department of Maternofetal Medicine, Genetics and Reproduction, Institute of Biomedicine of Seville (IBIS), University Hospital Virgen del Rocío/CSIC/University of Seville, Seville, Spain.

[2]Centre for Biomedical Network Research on Rare Diseases (CIBERER) Seville, Spain.

[3]Endocrine Surgery Unit. General Surgery Department, University Hospital Virgen del Rocío, Seville, Spain.

Corresponding author: Salud Borrego, PhD, MD, Department of Maternofetal Medicine, Genetics and Reproduction, IBIS, University Hospital Virgen del Rocío/CSIC/University of Seville. Av. Manuel Siurot s/n, 41013, Seville, Spain. Phone: +34-955312641. Centre for Biomedical Network Research on Rare Diseases (CIBERER), Seville, 41013, Spain. salud.borrego.sspa@juntadeandalucia.es.

Suppplementary Table 1: All 84 lncRNAs contained on arrays.

Position	UniGene	GenBank	Symbol	Description
A01	N/A	ENST00000437930	ACTA2-AS1	ACTA2 antisense RNA1
A02	N/A	ENST00000460833	ADAMTS9-AS2	ADAMTS9 antisense RNA 2
A03	N/A	ENST00000608442	AFAP1-AS1	AFAP1 antisense RNA 1
A04	N/A	ENST00000601203	AIRN	Antisense of IGF2R non-protein coding RNA
A05	N/A	NR_047671	BANCR	BRAF-activated non-protein coding RNA
A06	Hs.24611	NR_024049	BCAR4	Breast cancer anti-estrogen resistance 4
A07	N/A	NR_103783	BLACAT1	Bladder cancer associated transcript 1 (non-protein coding)
A08	N/A	ENST00000604200	CAHM	Colon adenocarcinoma hypermethylated (non-protein coding
A09	N/A	ENST00000413862	CBR3-AS1	CBR3 antisense RNA 1
A10	N/A	ENST00000500112	CCAT1	Colon cancer associated transcript 1 (non-protein coding)
A11	N/A	NR_109834	CCAT2	Colon cancer associated transcript 2 (non-protein coding)
A12	N/A	ENST00000421632	CDKN2B-AS1	CDKN2B antisense RNA 1
B01	N/A	ENST00000501177	CRNDE	Colorectal neoplasia differentially expressed (non-protein coding)
B02	N/A	NR_002733	DGCR5	DiGeorge syndrome critical region gene 5 (non-protein coding)
B03	Hs.547964	NR_002612	DLEU2	Deleted in lymphocytic leukemia 2 (non-protein coding)
B04	Hs.34969	NR_015448	DLX6-AS1	DLX6 antisense RNA 1
B05	Hs.312592	NR_002791	EMX2OS	EMX2 opposite strand (non-protein coding
B06	N/A	ENST00000418855	FTX	FTX transcript, XIST regulator (non-protein coding)
B07	N/A	ENST00000419650	GACAT1	Gastric cancer associated transcript 1 (non-protein coding)
B08	Hs.736055	NR_002578	GAS5	Growth arrest-specific 5 (non-protein coding)
B09	N/A	NR_044995	GAS6-AS1	GAS6 antisense RNA 1
B10	Hs.122718	NR_002785	GNAS-AS1	GNAS antisense RNA 1
B11	Hs.533566	NR_002196	H19	H19, imprinted maternally expressed transcript (non-protein coding)
B12	Hs.61435	NR_003679	HAND2-AS1	Nbla00301
C01	N/A	NR_045680	HEIH	Hepatocellular carcinoma up-regulated EZH2-associated long non-coding RNA
C02	N/A	ENST00000557544	HIF1A-AS1	HIF1A antisense RNA 1 [Source:HGNC Symbol;Acc:43014]

C03	N/A	NR_045406	HIF1A-AS2	HIF1A antisense RNA 2
C04	Hs.612351	ENST0000043333	HNF1A-AS1	HNF1A antisense RNA 1 (non-protein coding)
C05	Hs.197076	NR_003716	HOTAIR	Hox transcript antisense RNA (non-protein coding)
C06	N/A	ENST00000425358	HOTAIRM1	HOXA transcript antisense RNA, myeloid-specific 1
C07	N/A	ENST00000421733	HOTTIP	HOXA distal transcript antisense RNA
C08	Hs.587427	NR_002795	HOXA11-AS	HOXA11 antisense RNA 1 (non-protein coding)
C09	N/A	ENST00000517550	HOXA-AS2	HOXA cluster antisense RNA 2
C10	N/A	ENST00000503668	HULC	Hepatocellular carcinoma up-regulated long non-coding RNA
C11	N/A	NR_023915	IPW	Imprinted in Prader-Willi syndrome (non-protein coding)
C12	N/A	KC469579	JADRR	JADE1 adjacent regulatory RNA
D01	Hs.741312	NR_002728	KCNQ1OT1	KCNQ1 overlapping transcript 1 (non-protein coding)
D02	N/A	ENST00000407852	KRASP1	Kirsten rat sarcoma viral oncogene homolog pseudogene 1
D03	Hs.652166	NR_024204	LINC00152	Non-protein coding RNA 152
D04	N/A	NR_001558	LINC00261	Long intergenic non-protein coding RNA 261
D05	Hs.433151	NR_024065	LINC00312	Non-protein coding RNA 312
D06	N/A	NR_046189	LINC00538	Long intergenic non-protein coding RNA 538
D07	Hs.606465	NR_024480	LINC00887	Hypothetical LOC100131551
D08	N/A	ENST00000412141	LINC00963	Long intergenic non-protein coding RNA 963
D09	N/A	ENST00000594200	LINC01233	Long intergenic non-protein coding RNA 1233
D10	N/A	ENST00000510694	LINC01234	Long intergenic non-protein coding RNA 1234
D11	N/A	GU228577	LSINCT5	Long stress-induced non-coding transcript 5
D12	N/A	ENST00000511918	LUCAT1	Lung cancer associated transcript 1 (non-protein coding)
E01	Hs.642877	NR_002819	MALAT1	Metastasis associated lung adenocarcinoma transcript 1 (non-protein coding
E02	Hs.654863	NR_002766	MEG3	Maternally expressed 3 (non-protein coding)
E03	Hs.697120	NR_001458	MIR155HG	MIR155 host gene (non-protein coding)
E04	Hs.652877	NR_027349	MIR17HG	MiR-17-92 cluster host gene (non-protein coding)
E05	N/A	ENST00000304425	MIR31HG	MIR31 host gene (non-protein coding)
E06	Hs.326728	NR_027148	MIR7-3HG	Non-protein coding RNA 306
E07	N/A	ENST0000041980	MRPL23-AS1	MRPL23 antisense RNA 1
E08	N/A	NR_102270	NAMA	Non-protein coding RNA, associated with MAP kinase pathway and growth arrest
E09	Hs.559259	NR_003108	NBR2	Neighbor of BRCA1 gene 2 (non-protein coding)
E10	N/A	NR_028272	NEAT1	Nuclear paraspeckle assembly transcript 1 (non-protein coding)

E11	N/A	ENST00000517270	NRON	Non-protein coding RNA, repressor of NFAT
E12	N/A	NR_109836	PANDAR	Promoter of CDKN1A antisense DNA damage activated RNA
F01	Hs.663766	NR_015342	PCA3	
F02	N/A	ENST00000519319	PCAT1	Prostate cancer associated transcript 1 (non-protein coding)
F03	Hs.546994	NR_002769	PCGEM1	Prostate-specific transcript 1 (non-protein coding)
F04	N/A	ENST0000044559	POU5F1P5	POU class 5 homeobox 1 pseudogene 5
F05	N/A	ENST00000519282	PRNCR1	Prostate cancer associated non-coding RNA 1
F06	N/A	AK023948	PTCSC1	Papillary thyroid carcinoma susceptibility candidate 1 (non-protein coding)
F07	N/A	ENST0000055613	PTCSC3	Papillary thyroid carcinoma susceptibility candidate 3 (non-protein coding)
F08	Hs.493716	NR_023917	PTENP1	Phosphatase and tensin homolog pseudogene 1
F09	Hs.675281	NR_003367	PVT1	Pvt1 oncogene (non-protein coding)
F10	N/A	ENST0000036346	RMRP	RNA component of mitochondrial RNA processing endoribonuclease
F11	N/A	NR_024037	RMST	Rhabdomyosarcoma 2 associated transcript (non-protein coding)
F12	N/A	ENST00000455390	RPS6KA2-AS1	RPS6KA2 antisense RNA 1
G01	N/A	NR_038108	SNHG16	Small nucleolar RNA host gene 16 (non-protein coding)
G02	N/A	AK024556	SPRY4-IT1	SPRY4 intronic transcript 1 (non-protein coding)
G03	N/A	NR_002190	SUMO1P3	SUMO1 pseudogene 3
G04	N/A	ENST00000363312	TERC	Telomerase RNA component
G05	N/A	ENST00000431460	TRERNA1	Translation regulatory long non-coding RNA 1
G06	Hs.529901	NR_003255	TSIX	TSIX transcript, XIST antisense RNA (non-protein coding)
G07	Hs.554829	NR_002323	TUG1	Taurine upregulated 1 (non-protein coding)
G08	N/A	ENST00000466156	TUSC7	Tumor suppressor candidate 7 (non-protein coding)
G09	Hs.644234	NR_015379	UCA1	Urothelial cancer associated 1 (non-protein coding)
G10	Hs.567499	NR_023920	WT1-AS	WT1 antisense RNA (non-protein coding)
G11	Hs.529901	NR_001564	XIST	X (inactive)-specific transcript (non-protein coding)
G12	Hs.356766	NR_003604	ZFAS1	ZNFX1 antisense RNA 1
H01	Hs.520640	NM_001101	ACTB	Actin, beta
H02	Hs.534255	NM_004048	B2M	Beta-2-microglobulin
H03	Hs.546285	NM_001002	RPLP0	Ribosomal protein, large, P0
H04	N/A	NR_001445	RN7SK	RNA, 7SK small nuclear
H05	N/A	NR_002907	SNORA73A	Small nucleolar RNA, H/ACA box 73A
H06	N/A	SA_00105	HGDC	Human Genomic DNA Contamination

H07	N/A	SA_00104	RTC	Reverse Transcription Control
H08	N/A	SA_00104	RTC	Reverse Transcription Control
H09	N/A	SA_00104	RTC	Reverse Transcription Control
H10	N/A	SA_00103	PPC	Positive PCR Control
H11	N/A	SA_00103	PPC	Positive PCR Control
H12	N/A	SA_00103	PPC	Positive PCR Control
