# Supplementary material for: LncRNA LUCAT1 as a novel prognostic biomarker for patients with papillary thyroid cancer
Source: Sci Rep. 2019 Oct 7;9:14374. doi: 10.1038/s41598-019-50913-7 (PMC6779763; doi:10.1038/s41598-019-50913-7)
Supplement: Supplementary file 3 — Supplementary Table 2 [file 41598_2019_50913_MOESM3_ESM.pdf]

LncRNA *LUCAT1* as a novel prognostic biomarker for patients with papillary thyroid cancer.

LncRNA LUCAT1 as a novel prognostic biomarker for patients with papillary thyroid cancer.

Luzón Toro B[1,2], Fernández RM[1,2], Martos-Martínez JM[3], Rubio-Manzanares-Dorado M[3], Antiñolo G[1,2], Borrego S[1,2].

[1]Department of Maternofetal Medicine, Genetics and Reproduction, Institute of Biomedicine of Seville (IBIS), University Hospital Virgen del Rocío/CSIC/University of Seville, Seville, Spain.

[2]Centre for Biomedical Network Research on Rare Diseases (CIBERER) Seville, Spain.

[3]Endocrine Surgery Unit. General Surgery Department, University Hospital Virgen del Rocío, Seville, Spain.

Corresponding author: Salud Borrego, PhD, MD, Department of Maternofetal Medicine, Genetics and Reproduction, IBIS, University Hospital Virgen del Rocío/CSIC/University of Seville. Av. Manuel Siurot s/n, 41013, Seville, Spain. Phone: +34-955312641. Centre for Biomedical Network Research on Rare Diseases (CIBERER), Seville, 41013, Spain. salud.borrego.sspa@juntadeandalucia.es.

Supplementary Table 2: Knockdown of human *LUCAT1*: Silencing assays performed on all cell lines transfected at 24, 48 and 72 h with siRNA 1 and siRNA 2 at 5 and 20 µm. *LUCAT1* expression levels were determined by qRT-PCR, using GAPDH as internal control. All reactions were performed in triplicate.

**BCPAP_24h**							
GAPDH	**Ct**	**AVERAGE**	**ΔΔCt**	**2exp-ΔΔCt**	**Silencing rate (%)**	**t student**	**Standard Error**
si-NC	16,2678	16,432375	0	1			
siRNA1_5µM	15,2809	15,71485	0	1			
siRNA1_20µM	15,762	15,80115	0	1			
siRNA2_5µM	16,02	15,875	0	1			
siRNA2_20µM	16,47	16,41	0	1			
LUCAT1							
si-NC	25,9255	25,517025	0	1			0
siRNA1_5µM	26,7512	26,631175	1,831675	0,280938256	71,91	0,021	0,140
**siRNA1_20µM**	28,8136	27,74805	2,86225	0,137523493	**86,25**	0,002	0,270
siRNA2_5µM	27,77	27,82	0,33	0,79	21,00		0,049
siRNA2_20µM	27,19	26,7	-0,25	1,19	-19,00		0,150

**TPC1_24h**							
GAPDH	**Ct**	**AVERAGE**	**ΔΔCt**	**2exp-ΔΔCt**	**Silencing rate (%)**	**t student**	**Standard Error**
si-NC	18,354	18,35	0	1			
siRNA1_5µM	16,204	17,50	0	1			
siRNA1_20µM	16,167	16,26	0	1			
siRNA2_5µM	16,234	16,69	0	1			
siRNA2_20µM	17,499	17,38	0	1			
LUCAT1							
si-NC	29,097	29,0935	0	1			0
siRNA1_5µM	28,282	29,74725	1,54945	0,341640283	65,84	0,019	0,281
**siRNA1_20µM**	28,714	28,61825	1,6667	0,314972985	**68,50**	0,053	0,050

siRNA2_5μM	28,097	28,67525	1,2927	0,408186394	59,18	0,069	0,090
siRNA2_20μM	29,613	28,27875	0,20745	0,866066674	13,39	0,224	0,215

8505c_24h

GAPDH	**Ct**	**AVERAGE**	**ΔΔCt**	**2exp-ΔΔCt**	**Silencing rate (%)**	**t student**	**Standard Error**
si-NC	14,739	14,876	0	1			
siRNA1_5μM	14,645	14,505	0	1			
siRNA1_20μM	15,333	15,4635	0	1			
siRNA2_5μM	15,171	15,3535	0	1			
siRNA2_20μM	15,117	14,811	0	1			
LUCAT1							
si-NC	26,137	25,4605	0	1			0
siRNA1_5μM	28,767	28,2935	2,2455	0,210880851	85,97	0,048	0,25814349
**siRNA1_20μM**	28,295	28,578	2,64	0,160428237	**88,50**	0,025	0,28276974
siRNA2_5μM	28,605	27,75325	2,35775	0,195095175	79,60	0,022	0,23523688
siRNA2_20μM	29,5	28,01225	2,7015	0,153733129	83,00	0,042	0,26458592
